# Comparison of spectral-domain optical coherence tomography for intra-retinal layers thickness measurements between healthy and diabetic eyes among Chinese adults

**DOI:** 10.1371/journal.pone.0177515

**Published:** 2017-05-11

**Authors:** Shu-ting Li, Xiang-ning Wang, Xin-hua Du, Qiang Wu

**Affiliations:** 1 Department of Ophthalmology, Shanghai Jiaotong University Affiliated Sixth People’s Hospital, Shanghai, China; 2 Shanghai Key Laboratory of Diabetes Mellitus, Shanghai, China; Massachusetts Eye & Ear Infirmary, Harvard Medical School, UNITED STATES

## Abstract

**Purpose:**

To compare intra-retinal layer thickness measurements between eyes with no or mild diabetic retinopathy (DR) and age-matched controls using Spectralis spectral-domain optical coherence tomography (SD-OCT).

**Methods:**

Cross-sectional observational analysis study. High-resolution macular volume scans (30° * 25°) were obtained for 133 type 2 diabetes mellitus (T2DM) patients with no DR, 42 T2DM patients with mild DR and 115 healthy controls. The mean thickness was measured in all 9 Early Treatment Diabetic Retinopathy Study (ETDRS) sectors for 8 separate layers, inner retinal layer (IRL), outer retinal layer (ORL) and total retina (TR), after automated segmentation. The ETDRS grid consisted of three concentric circles of 1-, 3-, and 6-mm diameter. The superior, inferior, temporal, and nasal sectors of the 3- and 6-mm circles were respectively designated as S3, I3, T3, and N3 and S6, I6, T6, and N6. Linear regression analyses were conducted to evaluate the associations between the intra-retinal layer thicknesses, age, diabetes duration, fasting blood glucose and HbA1c.

**Results:**

The mean age and duration of T2DM were 61.1 and 13.7 years, respectively. Although no significant differences in the average TR and ORL volumes were observed among the groups, significant differences were found in the volume and sectorial thicknesses of the inner plexiform layer (IPL), outer plexiform layer (OPL) and IRL among the groups. In particular, the thicknesses of the IPL (S3, T3, S6, I6 and T6 sectors) and the IRL (S6 sector) were decreased in the no-DR group compared with the controls (P < 0.05). The thickness of the OPL (S3, N3, S6 and N6 sectors) was thinner in the no-DR group than in mild DR (P < 0.05). The average IPL thickness was significantly negatively correlated with age and the duration of diabetes.

**Conclusion:**

The assessment of the intra-retinal layer thickness showed a significant decrease in the IPL and IRL thicknesses in Chinese adults with T2DM, even in the absence of visible microvascular signs of DR.

## Introduction

The number of adults diagnosed with diabetes mellitus (DM) in the world has almost quadrupled, from 108 million in 1980 to 422 million in 2014, due to population growth and an increase in prevalence as well as the size of the aging population [1]. Diabetic retinopathy (DR), as one of the most common microvascular complications of diabetes, remains the leading cause of blindness in the working-age population [2]. Based on a meta-analysis, the prevalence of DR in mainland China was 1.3% in the pooled general population and 23% among diabetic individuals [3]. A widely accepted pathogenesis of DR consists of abnormalities and microvasculopathy [4,5], and the early clinical signs of DR include microaneurysms and retinal microhemorrhages [6,7]. However, research on the pathogenesis of DR found that neuronal dysfunction and neurodegeneration are closely correlated with microvascular dysfunction and that neurovascular unit degeneration should be considered as an important component of the pathology of DR [8–10]. Evidence from the retinas of both diabetic donors and diabetic animal models has also shown that retinal neuronal cell degeneration or apoptosis occurs early in the course of diabetes [11–13].

Advances in the resolution, acquisition speed, signal-to-noise ratio (SNR) and analysis algorithms of spectral-domain optical coherence tomography (SD-OCT) have allowed for the distinction of subtle retinal layer differences that resemble histological alterations [14,15]. Automated or manual multilayer segmentation of the retina [16–19] in clinical practice is constantly increasing, and topographic thickness map measurements provide valuable information about retinal cell layers [20,21]. For example, certain studies have shown that ganglion cell layer (GCL) thickness reduction is associated with visual function and can predict disease severity in multiple sclerosis [22,23]. The integrity of the retina ganglion cell (RGC) is crucial for preserving visual function, and the retinal nerve fiber layer (RNFL), the GCL and the inner plexiform layer (IPL) consist of the axons, nuclei and dendrites of RGCs, respectively. In type 2 diabetes mellitus (T2DM) patients, however, data from several OCT studies have been conflicting. Jay Chhablani et al. [24] suggested that early thinning of the intra-retinal layer occurs in T2DM even before visible vascular signs of DR, whereas van Dijk et al. [25,26] reported that GCL thickness was significantly thinner only in patients with apparent microvascular DR lesions. Furthermore, Chen Y et al. [27] found that the ganglion cell inner plexiform layer (GCIPL) complex thickness in T2DM patients was not significantly different from that of controls. Most of the previously published studies have focused on the analysis of macular GCIPL thickness via Cirrus HD-OCT (Carl Zeiss Meditec, Dublin, CA, USA) using a ganglion cell analysis (GCA) algorithm [24,27–29] and intra-retinal layer thickness via 3D OCT-1000 (Topcon Corp, Tokyo, Japan) [25,26]. These studies quantified RGC loss in terms of reduction of the peripapillary RNFL or macular GCIPL complex thickness and seldom reported the macular RNFL, GCL or IPL thickness changes separately. The latest version of Spectralis SD-OCT (Heidelberg Engineering, Heidelberg, Germany) is now packaged with software that is capable of segmenting a number of intra-retinal layers. Furthermore, few investigations have used Spectralis SD-OCT to assess changes in the neural retina in T2DM in Chinese individuals.

Different OCT devices use different segmentation algorithms to measure different parameters, and both the definition of the retinal boundaries and normative values vary between manufacturers [30–32]. The present study was performed to evaluate changes in the thickness of the intra-retinal layers, and particularly neuroretinal alterations, in Chinese type 2 diabetes patients with no DR or with microaneurysms only (mild DR) measured in three Early Treatment Diabetic Retinopathy Study (ETDRS) subfields (the central fovea and inner and outer circle subfields) and 9 sectors using Spectralis SD-OCT with the latest auto-segmentation software (version 6.3.1.0).

## Methods

The study protocol was approved by the Institutional Review Board of Shanghai Sixth People’s Hospital and was registered in the Chinese clinical trial registry (http://www.chictr.org.cn/, Registration number: ChiCTR-OOC-16010160). All of the procedures followed the tenets of the Declaration of Helsinki, and informed consent was obtained from all of the subjects before participation in the study. Diabetic patients were recruited from Shanghai Diabetes Centre at Shanghai Jiaotong University Affiliated Sixth People’s Hospital between December 1, 2016, and January 31, 2017. Healthy controls were recruited from the medical examination center at the same hospital during the same period.

### Participant enrollment

All of the participants underwent a full ophthalmologic examination, including clinical history taking, visual acuity assessment, biomicroscopy of the anterior segment using a slit lamp, ophthalmoscopy of the posterior segment, fundus photography using a nonmydriatic fundus camera (TRC-NW300; Topcon, Tokyo, Japan) and Spectralis SD-OCT scans. One eye was selected as the analysis eye for each participant, and if both eyes met the eligibility criteria, the eye with the better visual acuity or lower refractive error was included. For each participant in this study, we collected certain general demographic details (i.e., age, gender) and ophthalmologic examination results (i.e., visual acuity, refractive error, SD-OCT images, and signal quality). For each diabetic patient, a detailed medical history, including diagnoses, medications, disease duration and laboratory tests, was recorded. We extracted the clinical data from recorded free-text clinical notes and inpatient medical records.

All subjects (1) were aged 18 years or older, (2) had a log MAR visual acuity better than 0.5 log units, and (3) had a refractive error within -6.00 diopters or +3 diopter-equivalent spheres. Whether T2DM was coupled with no DR or mild DR was evaluated by a retinal specialist through indirect fundoscopy and fundus photography. Mild DR was defined as microaneurysms only, according to the International Clinical Diabetic Retinopathy Disease Severity Scale [33]. Age-matched control subjects with no DM and no ocular disease were recruited.

The exclusion criteria were as follows: (1) unable to provide informed consent or to undergo a complete examination; (2) pregnant or nursing; (3) presenting with a disease affecting retinal function, including glaucoma, advanced cataracts or significant media opacity, or with other serious systemic disease; (4) the presence of diabetic macular edema (DME); and (5) having undergone previous intraocular surgery.

### Spectral-domain optical coherence tomography scan protocol and analysis

All scans were obtained in a dark room without pupil dilation by a single retinal specialist. Spectralis SD-OCT uses a confocal laser scanning ophthalmoscope with a wavelength of 870 nm and an infrared reference image to obtain images, and it incorporates an automatic real-time (ART) eye tracker to eliminate motion artifacts. The focus and polarization settings were adjusted to achieve the best-quality image. Two consecutive replications of a 30° * 25° (9.1 * 7.6 mm) volume scan (ART mean of 9 [34] and high-speed model utilizing 512 A-scans per B-scan) were obtained. Subsequently, an operator reviewed all of the B-scans, verified that the images were centered on the fovea, and corrected any improper segmentation lines manually. The replicate scan with the highest image quality was selected for analysis. Eyes with more than three B-scans of poor quality or with improper segmentation were excluded from the analyses. Quality scores for the scans were expressed as an SNR in decibels (dB) over a range from 0 (poor quality) to 40 (excellent quality), and scans that scored greater than 20 dB were considered high quality. Only images that scored greater than 20 were included.

**Fig 1:**
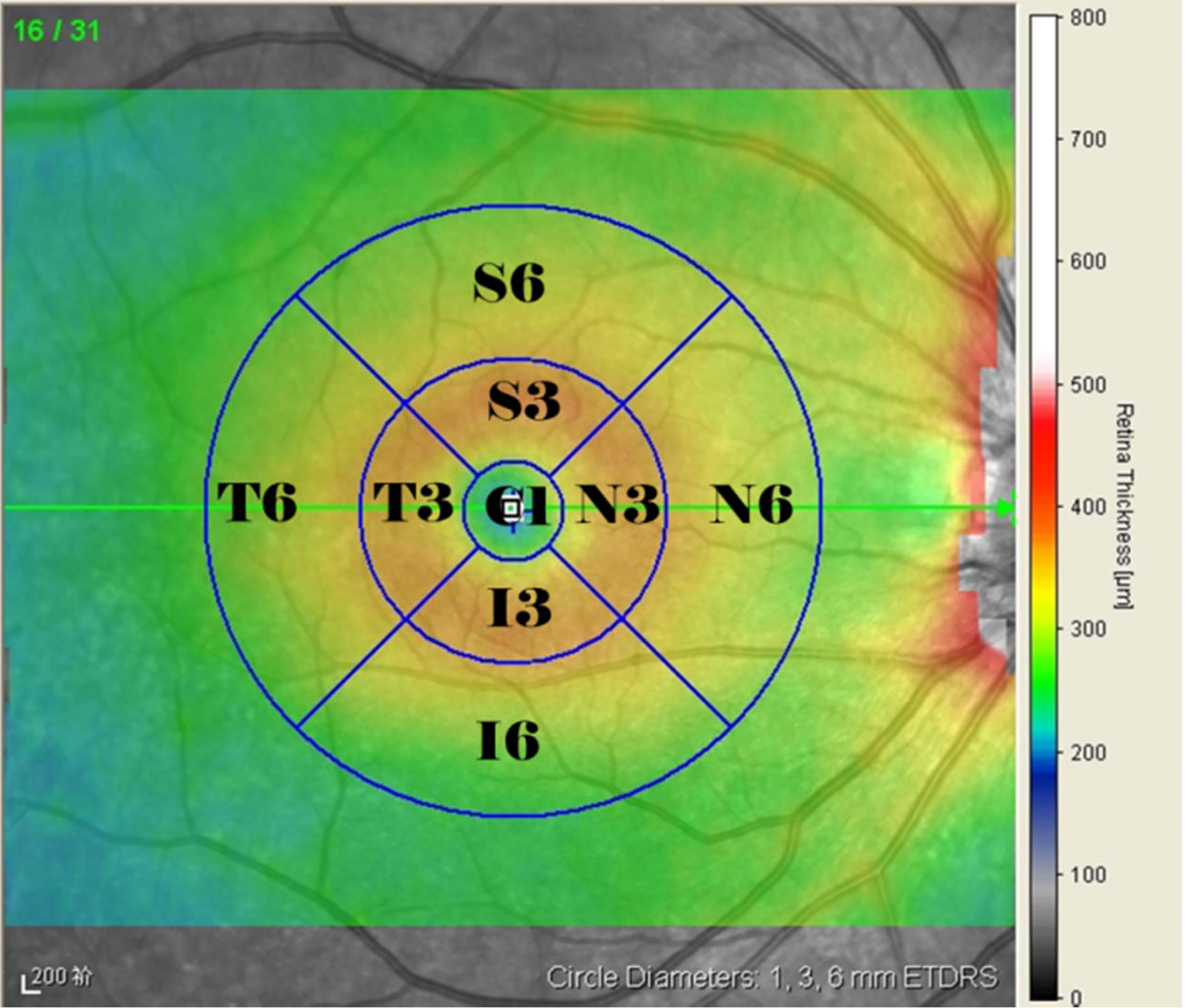
Representative Spectralis SD-OCT scans with macular thickness map (ETDRS grid). C1: the central fovea subfield/sector; S3, I3, T3, N3: superior, inferior, temporal, and nasal sectors, respectively, of the inner circle subfield between 1 and 3 mm; S6, I6, T6, N6: superior, inferior, temporal, and nasal sectors, respectively, of the outer circle subfield between 3 and 6 mm.

The ETDRS subfield measurements were performed using the inbuilt Spectralis mapping software, in which measurements are automatically averaged across each of the following subfields and sectors: the central fovea subfield within the inner 1-mm-diameter circle; the inner circle subfield between the inner and middle 3-mm-diameter circles; and the outer circle subfield between the middle and outer 6-mm-diameter circles. Both the inner and the outer circles were sectioned into superior, nasal, inferior, and temporal quadrants (see Fig 1).

**Fig 2:**
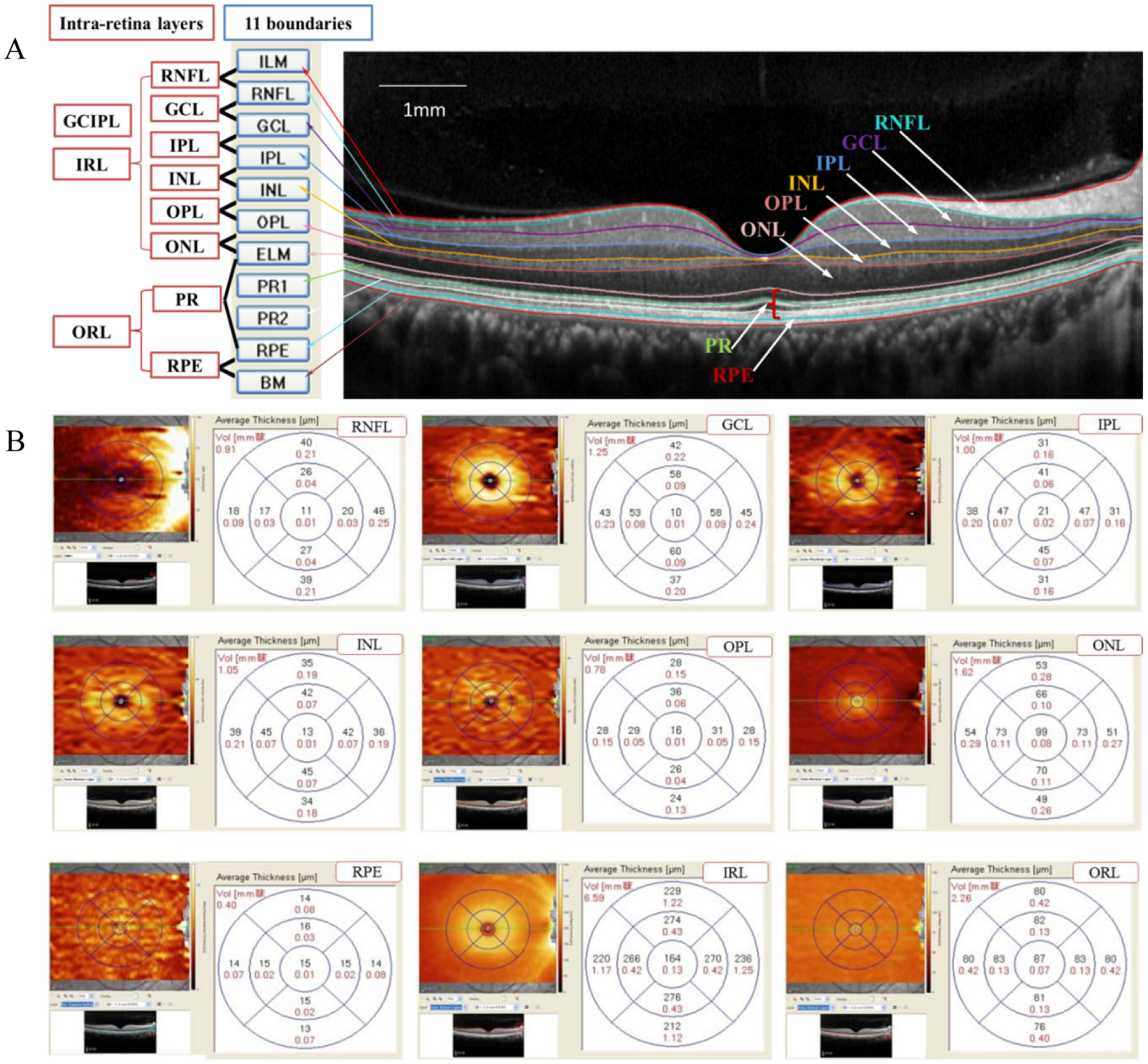
Representation of intra-retinal layer segmentation and thickness measurement. **(A):** Segmentation of the retinal boundaries in a normal eye by Spectralis SD-OCT: 1 = inner limiting membrane (ILM); 2 = retinal nerve fiber layer (RNFL); 3 = ganglion cell layer (GCL); 4 = inner plexiform layer (IPL); 5 = inner nuclear layer (INL); 6 = outer plexiform layer (OPL); 7 = outer limiting membrane (OLM); 8 = myoid zone of the photoreceptor layer (PR1); 9 = ellipsoid component of the photoreceptor layer (PR2); 10 = retinal pigment epithelium (RPE); and 11 = Bruch’s membrane. **(B):** Representative 9 intra-retinal layer measurements within a standard ETDRS grid: average volume and thickness measurements for the fovea subfield, inner and outer circle subfields and 9 sectors.

Segmentation of the retinal layers was performed on a horizontal macular volume scan using new auto-segmentation software (version 6.3.1.0; Segmentation Technology; Heidelberg Engineering, Inc.), which automatically separated the retina into 11 boundaries **(**Fig 2A**)** The macular volume of each layer was recorded in cubic millimeters. The averaged thicknesses of 3 circles and 9 sectors of the following layers were recorded in microns **(**Fig 2B**)**: the RNFL, GCL, IPL, inner nuclear layer (INL), outer plexiform layer (OPL), outer nuclear layer (ONL), retinal pigment epithelium (RPE), inner retinal layer (IRL), outer retinal layer (ORL) and total retina (TR). Because many researchers have utilized the GCIPL thickness and photoreceptor layer (PR) thickness [35–37], we also included these parameters in our analysis. The GCIPL and PR thicknesses were measured as the distances between the RNFL and IPL and between the internal limiting membrane (ILM) and RPE, respectively. The Spectralis system sets the outer retinal threshold line as Bruch’s membrane, so the TR thickness was measured as the vertical distance from the ILM to Bruch’s membrane.

### Statistical analysis

All of the data analyses were performed using SPSS software, version 19.0 (IBM Corporation, Chicago, IL, USA). The results are expressed as the mean ± standard deviation (SD) for continuous variables and as a percentage for categorical variables. One-way analysis of variance (ANOVA) with Bonferroni’s post hoc tests, independent t tests or Mann-Whitney U tests was performed, as appropriate, to compare the continuous variables. Additionally, χ^2^ tests were used to compare the proportions among groups. A linear regression model was also used to assess the relationship between the average intra-retinal layer thicknesses and variables including age, the duration of diabetes, fasting blood glucose (FBG), and HbA1c. All P values reported were two tailed. A type I error level of 0.05 was used to assess statistical significance.

## Results

After screening according to the inclusion/exclusion criteria, a total of 290 participants (115 healthy controls and 175 diabetes patients; 152 men and 138 women) were included in this study, of whom 115 were classified as the control group, 133 were classified as having T2DM with no DR (no-DR group), and 42 were classified as having mild DR. The demographic characteristics of the study participants are shown in Table 1.

**Table 1:** Demographic and clinical characteristics of the study participants.

Characteristics	control 115 eyes	T2DM 175 eyes	P1 value	no- DR 133 eyes	mild DR 42 eyes	P2 value
**Number**	115	175	**-**	133	42	-
**Age, y**	58.6 ± 13	61.1 ± 11	0.087	60.8 ± 11	62.1 ± 11	0.175
**Gender(male/female)**	62/53	90/85	0.679	68/65	22/20	0.909
**Laterality(right/left)**	57/58	94/81	0.431	73/60	21/21	0.598
**Visual acuity, log MAR**	0.05 ± 0.10^§^	0.06 ± 0.11	0.688	0.05 ± 0.11^#^	0.09 ± 0.11^#§^	0.037
**Refractive error, diopters**	-0.96 ± 2.20	-0.90± 2.25	0.331	-0.91 ± 2.33	-0.70 ± 2.23	0.810
**Signal quality, dB**	26 ± 2.4	24 ± 2.9	0.146	25 ± 2.7	25 ± 3.2	0.198
**Duration of diabetes, y**	-	13.7 ± 8.4	-	13.0 ± 8.2	15.7 ± 8.4	-
**FBG,mmoL/L**	-	8.2 ± 2.4	-	8.1 ± 2.4	8.7 ± 2.4	-
**HbA1c, %**	-	8.3 ± 1.7	-	8.2 ± 1.7	8.6 ± 1.6	-

All of the values are presented as the mean ± SD. P1 values: significant difference in Student’s t test between the control group and both DM groups. P2 values: significant difference by ANOVA among the control, no-DR, and mild DR groups. The symbols “#, §” indicate significant differences (P < 0.05) between groups using Bonferroni’s post hoc test. FBG: fasting blood glucose; -: not performed.

The mean ages of the control group and both diabetes groups were 58.6 ± 13 years (range 28–85) and 61.1 ± 11 years (range 30–85), respectively. There was no significant difference in terms of age (P = 0.087), sex (P = 0.679), laterality (P = 0.431), visual acuity (P = 0.688), mean spherical equivalent (P = 0.331) or scan quality (P = 0.146) between the control group and the two diabetes groups. ANOVA followed by Bonferroni’s post hoc test was performed to examine the differences among the three groups and indicated no statistically significant differences in any parameter except for visual acuity between the mild DR and controls (P = 0.041) and between the mild DR and no-DR (P = 0.032).

### Intra-retinal layer and macular thickness measurements

**Fig 3:**
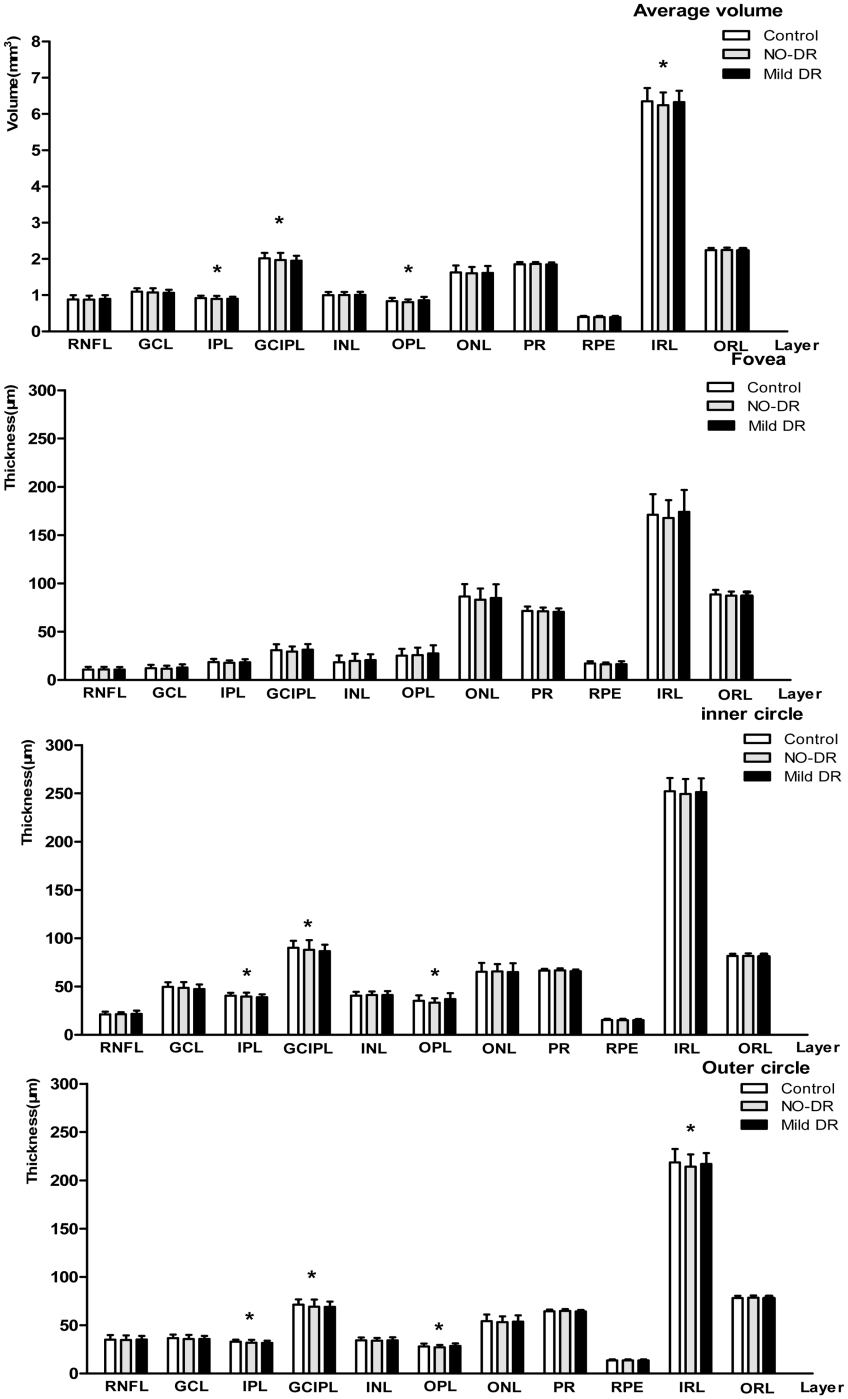
Intra-retinal layer volume and 3 subfields of ETDRS grid thicknesses. The character “*” indicates significant differences among the groups using ANOVA (P < 0.05). Retinal nerve fiber layer (RNFL), ganglion cell layer (GCL), ganglion cell and inner plexiform layer (GCIPL), inner plexiform layer (IPL), inner nuclear layer (INL), outer plexiform layer (OPL), outer nuclear layer (ONL), photoreceptor layer (PR), retinal pigment epithelium (RPE), inner retinal layer (IRL) and outer retinal layer (ORL).

Comparisons of the average intra-retinal layer volumes and the central fovea and inner and outer subfield thicknesses were performed among the 3 groups (Fig 3). Sectored analyses of each layer’s thickness in the ETDRS grid were shown in Tables 2–4. On average, the inner subfields were thicker than the outer subfields, and the nasal sectors were thicker than the temporal sectors. Although no significant differences in the average volumes of the TR and ORL were observed among the groups, the average IRL volume was significantly reduced in the eyes of the no-DR group compared with the controls: 6.25 ± 0.36 mm versus 6.35 ± 0.36 mm (Bonferroni’s post hoc test, P = 0.038). The average volumes of the IPL and GCIPL in the control, no-DR, mild DR groups (mm) were respectively 0.92 ± 0.06, 0.89 ± 0.07, and 0.89 ± 0.06 (P = 0.010) and 2.01± 0.15, 1.96 ± 0.20, and 1.95± 0.13 (P = 0.030). Moreover, the average outer subfield thicknesses of the IPL and GCIPL were significantly thinner in diabetic eyes with no-DR than in the controls, and a significant difference was found in the inner and outer subfield thicknesses of the OPL among the groups.

**Table 2:** RNFL, GCL, IPL, and GCIPL volumes and thicknesses in ETDRS grid for each group.

	RNFL			GCL			IPL			GCIPL		
	Control	no-DR	mild DR	Control	no-DR	mild DR	Control	no-DR	mild DR	Control	no-DR	mild DR
Average volume,mm^3^	0.88 ± 0.11	0.88 ± 0.11	0.90 ± 0.10	1.10 ± 0.09	1.07 ± 0.11	1.06 ± 0.08	0.92 ± 0.06*	0.89 ± 0.07*	**0.89 ± 0.06**	2.01± 0.15*	1.96 ± 0.20*	**1.95± 0.13**
EDTRS grid, μm												
**Central fovea subfield**	10.9 ± 2.7	11.0 ± 2.6	10.9 ± 2.5	12.2 ± 3.4	11.9 ± 3.1	12.8 ± 3.4	18.6 ± 3.2	17.8 ± 2.6	18.4 ± 3.1	30.8 ± 6.2	29.6 ± 5.3	31.1 ± 5.9
**Inner 3-mm subfield**	21.1 ± 2.9	21.2 ± 2.2	21.6 ± 3.3	49.6 ± 4.9	48.5 ± 6.1	47.5 ± 4.5	**40.5 ± 3.0**	**39.5 ± 4.2**	**39.0 ± 3.0**	**90.1 ± 7.3**	**88.0 ± 9.3**	**86.5 ± 6.9**
Superior	23.5 ± 3.8	23.1 ± 3.1	23.3 ± 5.3	52.1 ± 5.2	51.0 ± 5.7	49.8 ± 5.1	40.6 ± 3.2*^§^	39.4 ± 3.9*	**38.7 ± 3.3**^**§**^	92.7 ± 7.9*	90.4 ± 9.3*	**88.4 ± 7.5**
Inferior	23.9 ± 3.8	24.0 ± 3.4	24.5 ± 4.6	50.4 ± 5.2	49.0 ± 6.7	48.1 ± 5.3	39.7 ± 3.5	39.1 ± 4.6	38.2 ± 3.6	90.0 ± 7.6	88.0 ± 11.1	86.3 ± 8.2
Temporal	**17.2 ± 2.1**^**§**^	**17.5 ± 2.0**^**#**^	**18.5 ± 3.2**^**#§**^	48.3 ± 6.0	48.0 ± 6.9	46.5 ± 4.8	40.9 ± 3.5*	39.6 ± 5.0*	**39.9 ± 3.6**	89.2 ± 8.5	87.9 ± 11.0	86.4 ± 7.4
Nasal	19.9 ± 3.0	20.2 ± 3.1	20.6 ±4.7	47.5 ± 6.0	46.0 ± 7.4	45.5 ± 5.5	**41.0 ± 3.9**	**40.1 ± 4.4**	**39.2 ± 3.5**	88.4 ± 8.6	86.1 ± 11.1	84.8 ± 8.1
**Outer 6-mm subfield**	35.0 ± 4.8	34.8 ± 4.6	35.3 ± 3.8	36.8 ± 3.6	35.8 ± 4.1	35.7 ± 3.4	32.9 ± 2.3*	31.9 ± 2.9*	**31.7 ± 2.3**	71.3 ± 5.6*	69.3 ± 7.2*	**69.1 ± 5.5**
Superior	37.8 ± 6.1	36.9 ± 5.6	37.7 ± 5.2	36.6 ± 3.7	35.6 ± 4.1	35.4 ± 3.4	29.6 ± 2.8*	28.6 ± 3.0*	**28.7 ± 2.7**	**66.3 ± 6.2**	**64.3 ± 7.0**	**64.1 ± 5.8**
Inferior	37.9 ± 7.1	37.7 ± 6.9	38.8 ± 4.8	33.1 ± 4.0	32.5 ± 4.2	32.8 ± 4.3	27.5± 3.0*	26.5 ± 3.0*	**26.7 ± 2.2**	60.6 ± 6.4	59.0 ± 6.9	59.5 ± 6.7
Temporal	**19.2 ± 2.6**^**§**^	**19.3 ± 2.3**	**20.3± 3.5**^**§**^	36.5 ± 4.5	35.3 ± 5.2	35.5 ± 4.3	33.3 ± 2.9*	32.3 ± 3.4*	**32.3 ± 3.0**	69.7 ±6.9	67.6 ± 7.8	67.8 ± 6.6
Nasal	45.1 ± 6.8	45.2 ± 7.4	44.0 ± 6.5	40.7 ± 4.3	39.8 ± 4.6	39.2 ± 4.1	31.4 ± 3.1	30.6 ± 3.6	30.9 ± 2.5	72.1 ± 7.0	70.4 ± 7.9	70.1 ± 6.1

All of the values are presented as the mean ± SD (μm). The inner subfield thickness is the average of the four parafoveal sectors, and the outer subfield thickness is the average of the four perifoveal sectors. Bold values indicate statistically significant differences among the three groups by ANOVA. The characters “*”, “§” and “#” indicate significant differences by Bonferroni’s post hoc test (P<0.05) between the no-DR and control groups, between the mild DR and control groups, and between the no-DR and mild DR groups, respectively. Retinal nerve fiber layer (RNFL), ganglion cell layer (GCL), inner plexiform layer (IPL), ganglion cell and inner plexiform layer (GCIPL).

**Table 3:** INL, OPL, ONL, and IRL volumes and thicknesses in ETDRS grid for each group.

	INL			OPL			ONL			IRL		
	Control	no-DR	mild DR	Control	no-DR	mild DR	Control	no-DR	mild DR	Control	no-DR	mild DR
Average volume, mm	0.99 ± 0.08	1.00 ± 0.08	1.00 ± 0.09	**0.84 ± 0.09**	**0.81 ± 0.08**^**#**^	**0.86 ± 0.09**^**#**^	1.63 ± 0.19	1.61 ± 0.17	1.61 ± 0.19	6.35 ± 0.36*	6.25 ± 0.36*	**6.32 ± 0.36**
EDTRS, μm												
**Central fovea subfield**	18.5 ± 6.9	19.8 ± 7.6	20.7 ± 6.0	25.4 ± 7.5	25.7 ± 7.8	27.6 ± 8.5	86.4 ± 12.6	83.1 ± 11.5	84.8 ± 14.3	171 ± 21	168 ± 19	174 ± 22
**Inner 3-mm subfield**	40.5 ± 4.1	41.2 ± 3.7	41.1 ± 4.1	35.3 ± 5.5*	33.8 ± 4.6*^#^	**37.0 ± 6.2**^**#**^	65.5 ± 8.8	65.8 ± 7.8	65.0 ± 9.2	252 ± 14	250 ± 16	251 ± 14
Superior	41.1 ± 4.7	41.6 ± 4.4	41.6 ± 4.7	**35.4 ± 10.7**	**34.0 ± 8.2**^**#**^	**39.0 ± 12.0**^**#**^	64.7 ± 13.2	65.2 ± 11.0	63.4 ± 14.2	257 ± 15	254 ± 17	255 ± 13
Inferior	41.0 ± 4.7	41.8 ± 4.5	41.0 ± 5.1	35.7 ± 10.1	33.7 ± 8.9	35.5 ± 9.0	62.7 ± 15.0	62.8 ± 11.1	63.0 ± 13.5	253 ± 15	251 ± 17	251 ± 16
Temporal	38.1 ± 5.2	39.2 ± 4.3	38.8 ± 4.1	31.3 ± 6.2	31.9 ± 7.3	31.9 ± 6.7	69.2 ± 10.4	68.5 ± 9.2	70.8 ± 11.0	244 ± 15	241 ± 17	245 ± 15
Nasal	41.8 ± 5.5	42.1 ± 5.0	42.9 ± 5.9	**38.0 ± 10.3**	**35.6 ± 10.3**^**#**^	**41.7 ± 13.2**^**#**^	65.1 ± 14.8	66.6 ± 12.9	62.6 ± 15.1	254 ± 15	253 ± 16	254 ± 16
**Outer 6-mm subfield**	34.3 ± 3.0	34.0 ± 2.9	34.4 ± 3.2	**28.0 ± 3.0**	**27.3 ± 2.5**^**#**^	**28.6 ± 2.6**^**#**^	54.2 ± 6.6	53.3 ± 6.0	53.8 ± 6.4	219 ± 14*	215 ± 13*	**217 ± 11**
Superior	33.3 ± 3.5	33.4 ± 3.8	33.9 ± 4.2	**26.9 ± 4.0**	**26.0 ± 4.1**^**#**^	**26.8 ±2.9**^**#**^	57.3 ± 7.3	56.7 ± 6.7	56.5 ± 5.9	221 ± 17*	217 ± 13*	**220 ± 12**
Inferior	33.3 ± 4.4	32.9 ± 4.2	33.1 ± 3.9	27.2 ± 4.0	26.7 ± 4.1	26.8 ± 2.7	50.9 ± 8.8	49.3 ± 6.3	50.3 ± 8.4	209 ± 14	205 ± 14	208 ± 13
Temporal	34.2 ± 3.0	33.8 ± 2.8	33.6 ± 3.1	27.0 ± 3.2	26.4 ± 2.7	26.9 ± 2.7	56.0 ± 8.0	54.8 ± 5.9	56.4 ± 8.0	206 ± 16	203 ± 14	206 ± 14
Nasal	36.6 ± 3.8	36.0 ± 3.7	36.9 ± 4.9	**30.9 ± 5.1**	**30.1 ± 4.6**^**#**^	**32.8 ± 5.7**^**#**^	52.9 ± 8.9	52.4 ± 8.5	52.0 ± 8.7	237 ± 16	233 ± 16	234 ± 22

All of the values are presented as the mean ± SD (μm). The inner subfield thickness is an average of the four parafoveal sectors, and the outer subfield is an average of the four perifoveal sectors. Bold values indicate statistically significant differences among the three groups by ANOVA. The characters “*” and “#” indicate significant differences by Bonferroni’s post hoc test (P<0.05) between the no-DR and control groups, and between the no-DR and mild DR groups, respectively. INL: inner nuclear layer, OPL: outer plexiform layer (OPL), ONL: outer nuclear layer, IRL: inner retina layer

**Table 4:** PR, RPE, ORL, and TR volumes and thicknesses in ETDRS grid for each group.

	PR			RPE			ORL			TR		
	Control	no-DR	mild DR	Control	no- DR	mild DR	Control	no-DR	mild DR	Control	no-DR	mild DR
Average volume, mm	1.84 ± 0.05	1.85 ± 0.05	1.83 ± 0.04	0.40 ± 0.03	0.40 ± 0.03	0.39 ± 0.03	2.2 ± 0.1	2.2 ± 0.1	2.2 ± 0.1	8.6 ± 0.4	8.5 ± 0.4	8.6 ± 0.3
EDTRS, μm												
**Central fovea subfield**	71.6 ± 4.4	71.1 ± 4.0	70.6 ± 3.7	16.5 ± 2.4	16.2 ± 2.1	16.0 ± 2.0	88.4 ± 4.7	87.5 ± 4.6	87.0 ± 4.3	260 ± 20	255 ± 19	261 ± 22
**Inner 3-mm subfield**	66.4 ± 1.9	66.6 ± 2.1	66.0 ± 2.0	15.2 ± 1.4	15.1 ± 1.5	15.1 ± 1.4	81.7 ± 2.6	81.7 ± 2.8	81.1 ± 2.6	333 ± 15	331 ± 16	332 ± 15
Superior	66.3 ± 2.1	66.3 ± 2.3	65.7 ± 2.0	15.6 ± 1.8	15.6 ± 2.0	15.5 ± 1.8	81.9 ± 2.9	81.9 ± 3.1	81.2 ± 3.2	339 ± 16	336 ± 17	336 ± 14
Inferior	65.4 ± 2.0	65.7 ± 2.1	65.2 ± 1.6	14.8 ± 1.5	14.7 ± 1.7	14.8 ± 1.6	80.2 ± 2.8	80.4 ± 3.0	80.0 ± 2.7	334 ± 15	332 ± 18	332 ± 16
Temporal	66.8 ± 2.3	67.2 ± 2.5	70.8 ± 11.0	14.9 ± 1.3	14.6 ± 1.5	14.6 ± 1.4	81.8 ± 2.7	81.8 ± 2.9	81.0 ± 2.8	326 ± 16	323 ± 16	326 ± 16
Nasal	67.1 ± 2.3	67.3 ± 2.4	66.7 ± 2.0	15.6 ± 1.8	15.5 ± 1.7	15.4 ± 1.7	82.7 ± 3.0	82.8 ± 3.1	82.1 ± 3.0	337 ± 17	335 ± 17	336 ± 16
**Outer 6-mm subfield**	64.6 ± 1.8	64.9 ± 1.7	64.3 ± 1.7	13.6 ± 1.0	13.6 ± 1.0	13.5 ± 1.1	78.2 ± 2.2	78.6 ± 2.3	77.9 ± 2.3	297 ± 15	297 ± 14	295 ± 15
Superior	65.1 ± 1.9	65.3 ± 2.0	64.5 ± 1.9	14.0 ± 1.3	14.2 ± 1.4	13.8 ± 1.3	79.1 ± 2.5	79.5 ± 2.6	78.5 ± 2.5	300 ± 16	296 ± 14	298 ± 12
Inferior	63.5 ± 2.0	63.8 ± 1.9	63.3 ± 2.2	13.1 ± 1.1	13.1 ± 1.2	13.1 ± 1.2	76.6 ± 2.5	77.0 ± 2.6	76.5 ± 2.7	286 ± 15	282 ± 14	285 ± 13
Temporal	65.0 ± 2.3	65.4 ± 2.0	64.6 ± 1.8	13.6 ± 1.1	13.5 ± 1.1	13.5 ± 1.3	78.6 ± 2.7	78.9 ± 2.5	78.1 ± 2.5	285 ± 16	281 ± 14	284 ± 15
Nasal	64.8 ± 2.4	65.2 ± 1.7	64.8 ± 1.5	13.8 ± 1.5	13.6 ± 1.4	13.5 ± 1.5	78.5 ± 2.9	78.8 ± 2.4	78.2 ± 2.3	316 ± 16	312 ± 17	312 ± 22

All of the values are presented as the mean ± SD (μm). The inner subfield thickness is an average of the four parafoveal sectors, and the outer subfield is an average of the four perifoveal sectors. Bold values indicate statistically significant differences among the three groups by ANOVA. PR: photoreceptor layer, RPE: retinal pigment epithelium, ORL: outer retinal layer, TR: total retina

In quantitative analyses of the sectors, ANOVA with Bonferroni’s post hoc test indicated statistically significant differences (P < 0.05) in thickness in the following sectors of the retinal layers: the S3, I3, S6, I6, and T6 sectors of the IPL and the S6 sector of the IRL. Specifically, these thicknesses were thinner in patients with diabetes with no DR than in the controls. In addition, the T3 and T6 sectors of the RNFL were significantly thicker in eyes with mild DR compared with the controls, and the RNFL (T3 sector) and OPL (the S3, N3, S6 and N6 sectors) were significantly thicker in eyes with mild DR compared with eyes with no DR.

### Correlations and predictors of intra-retinal layer thicknesses

Table 5 illustrates the linear regression of the average IPL, GCIPL, and OPL thicknesses with parameters in diabetes. The duration of diabetes was significantly negatively correlated with the average IPL and GCIPL thicknesses, whereas no correlation was found with either glycaemia or HbA1c.

**Table 5:** Correlations and predictors of IPL, GCIPL, OPL thickness (linear regression).

Parameters	IPL		GCIPL		OPL	
	β	P	β	P	β	P
Age, y	**-0.33**	**<0.001**	**-0.34**	**<0.001**	**0.15**	**0.015**
Duration of diabetes, y	**-0.25**	**<0.001**	**-0.22**	**<0.001**	-0.04	0.512
FBG,mmoL/L	-0.04	0.498	-0.04	0.590	-0.12	0.132
HbA1c, %	-0.01	0.973	-0.02	0.843	0.03	0.677
RNFL thickness	**0.44**	**<0.001**	**0.47**	**<0.001**	0.04	0.525
GCL thickness	**0.92**	**<0.001**	**0.98**	**<0.001**	0.07	0.263
IPL thickness	**-**	**-**	**0.96**	**<0.001**	**0.20**	**0.001**
GCIPL thickness	**0.97**	**<0.001**	**-**	**-**	**0.13**	**0.034**
INL thickness	**0.42**	**<0.001**	**0.30**	**<0.001**	**0.27**	**<0.001**
OPL thickness	**0.20**	**0.002**	**0.13**	**0.034**	**-**	-
ONL thickness	0.04	0.187	0.04	0.537	**-0.25**	**<0.001**
PR thickness	0.01	0.804	0.04	0.569	0.02	0.755
RPE thickness	-0.05	0.732	-0.03	0.573	-0.01	0.900
IRL thickness	**0.81**	**<0.001**	**0.76**	**<0.001**	**0.25**	<0.001
ORL thickness	0.00	0.985	0.01	0.872	0.01	0.858
TR thickness	**0.78**	**<0.001**	**0.74**	**<0.001**	**0.24**	<0.001

Bold values indicate statistically significant relationships between parameters. β: Standardized Coefficients

**Fig 4:**
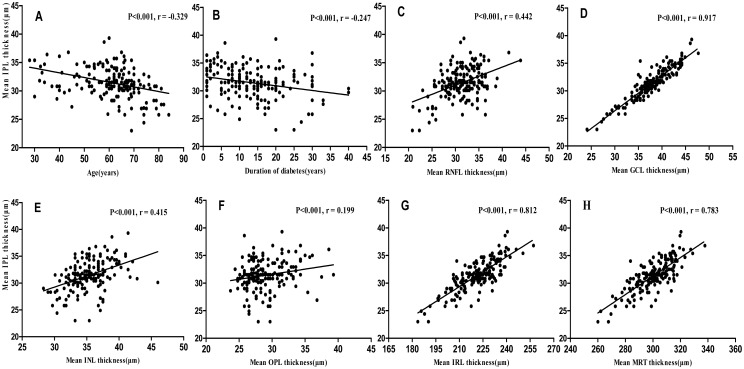
**A-H. Scatterplots for simple linear regression between the mean IPL thickness** and age (A), the duration of diabetes (B), the mean RNFL thickness (C), the mean GCL thickness (D), the mean INL thickness (E), the mean OPL thickness (F), the mean IRL thickness (G), and the mean TR thickness (H).

Fig 4 shows the relationships of the average IPL thickness with age, the duration of diabetes and other intra-retinal layer thicknesses.

## Discussion

Retinal layer segmentation and quantitative thickness measurements provide important information for disease detection and diagnosis. The current study presents the mean values of intra-retinal layer volumes and sectorial thicknesses and their distribution characteristics in Chinese adults with T2DM and healthy controls. We demonstrated that the newly available auto-segmentation technology of Spectralis SD-OCT could detect significant decreases in the thickness of the inner retina (IPL, GCIPL and OPL), which might reflect the gross anatomical loss of ganglion cell nuclei and dendrites, in the eyes of diabetic patients with no DR compared with healthy controls.

Several previously published studies have investigated total retinal thickness or isolated RNFL or GCIPL thickness in diabetic patients. Using a Topcon 3D OCT-1000, van Dijk et al. [25, 26] found that the pericentral area of the GCL and the peripheral area of the RNFL were significantly thinned in patients with mild DR, but not in diabetic patients with no DR, among a small number of subjects. Meanwhile, Chen Y et al. [27] and Demir et al. [38] demonstrated that the thicknesses of the RNFL and GCIPL in type 2 diabetes patients were lower than in healthy controls, but the results were not statistically significant. In contrast, Jay Chhablani et al. [24] reported early thinning of ganglion cell complex thickness using the Cirrus HD-OCT, and Vujosevic et al. [39] found a decrease in the RNFL thickness in type 2 diabetes patients who had no apparent microvascular signs of DR. Recently, Tavares Ferreira J et al. [40] found a thinner retina, with decreased thickness particularly in the inner layers, in diabetic patients without DR after 1 year of follow-up. Consistent with previous publications, the current study also found no significant differences in the TR and ORL thicknesses among the 3 groups, but significant thinning of the IRL, and particularly the IPL and GCIPL, was found in diabetic subjects without visible vascular signs of DR. Compared with previous studies, our study showed markedly diffuse thinning of the IPL in eyes in both the mild DR and the NO-DR groups using Spectralis SD-OCT intra-retinal layer thickness measurements, and Bonferroni’s post hoc test indicated a statistically significant difference between the diabetes patients with no DR and the control subjects. Although different SD-OCT instruments have different scanning processes, methods of identification of the outer retinal boundary lines and segmentation algorithms, Spectralis and the Cirrus HD-OCT instrument have similarities in retinal thickness measurement [30], and their ability to detect glaucoma damage is comparable[17]. In addition, results from our study supported and strengthened previous histological findings showing that IPL thinning (RGC degeneration begins with the dendrites) [41] and retinal amacrine neuronal dysfunction can be observed in diabetic patients [10,12].

It has recently been accepted that the pathogenesis of diabetes might involve a change in the retinal neurovascular unit [42]. This concept is supported by both experimental and clinical research suggesting that neural apoptosis and retinal neurodegeneration are found in the very early stages of DR [43,44]. It has also been demonstrated that glutamate accumulation, oxidative stress, inflammation, imbalances in retinal proapoptosis and survival signaling might be involved in the molecular mechanisms of neuronal damage during the development of DR [45,46].

Consistent with previous publications, the current study found that reduction of the thicknesses of the IPL and GCIPL was associated with age and with the duration of diabetes [47,48]. The parameters associated with a thinner IPL included a thinner RNFL, GCL, INL or TR and older age as well as a longer duration of diabetes. These statistically significant relationships among the average RNFL, GCL and IPL thicknesses are not surprising because these layers are closely related, consisting of the axons, nuclei and dendrites of RGCs, respectively. In particular, the OPL thickness gradually increases in patients with diabetes with the presence of microvascular DR lesions, likely due to deterioration of the blood-retinal barrier and the increased vascular permeability of diabetic eyes [49]. However, Oberwahrenbrock T [50] suggested that the reliability of OPL measurements was weak in general and needed further investigation before OPL thickness could be used as a reliable parameter.

It has been shown that SD-OCT image quality can influence retinal thickness measurements [51] and that the image data quality is affected by instrument reliability, measurement variability, media opacity, and eye movement and centering of the scans [52,53]. As widely acknowledged by clinicians, SD-OCT has shown excellent repeatability, with an infraclass correlation coefficient greater than 0.90 [17,54,55]. Ctori I et al. [56] also demonstrated that Spectralis SD-OCT segmentation software had excellent repeatability and reproducibility for each of eight individual retinal layer thickness measurements in healthy adults. However, the repeatability of GCIPL thickness measurements was lower in macular edema and atrophy patients [35]. The current study considered individual ocular biometry, including refractive error, and excluded patients with the presence of DME and other diseases affecting retinal function. Macular volumetric SD-OCT images were obtained using the ART eye tracker to eliminate motion artifacts, and we only selected images with good quality.

There were certain limitations to the current study. First, this was a cross-sectional study. Second, the FBG and HbA1c of the control group were unknown. Some healthy participants might have had subclinical diabetes. Third, the small sample size of the group with diabetes with mild DR could have limited potential associations. Fourth, we only included subjects with good visual acuity and OCT images with good quality. These limitations must be considered when interpreting the OCT data and retinal segmentation. Furthermore, there were differences between the groups beyond a reduction in thickness, which suggests that a further longitudinal study is needed to investigate the morphologic changes in different stages of DR.

In conclusion, the data revealed significant thickness alterations in the inner retinal layers, and particularly the IPL, GCIPL and OPL, in the eyes of Chinese adults with T2DM. Intra-retinal thickness measurement by auto-segmentation, rather than total retinal thickness measurement, may serve as a potential tool to identify intra-retinal layer thickness changes in eyes in the early stages of DR.

## Supporting information

S1 Table Data set. (XLSX)
